# Hepatic micro‐abscesses as an unusual initial presentation of systemic lupus erythematosus: A case report

**DOI:** 10.1002/ccr3.8586

**Published:** 2024-04-24

**Authors:** Sreethish Sasi, Mugahid Eltahir, Ezzeddin Ibrahim, Aishwariya Padmakumari, Jouhar Kolleri, Tasneem Abdallah, Ahmed Labib Shehatta, Nawal Hadwan, Hani Jaouni, Muna Al‐Maslamani

**Affiliations:** ^1^ Department of Infectious Diseases Communicable Diseases Center, Hamad Medical Corporation Doha Qatar; ^2^ Department of Critical Care Hamad Medical Corporation Doha Qatar; ^3^ Department of Rheumatology Hamad Medical Corporation Doha Qatar; ^4^ Department of Clinical Imaging Hamad Medical Corporation Doha Qatar

**Keywords:** autoimmune disease, case report, diagnostic challenges, hepatic micro‐abscesses, hepatic vasculitis, systemic lupus erythematosus

## Abstract

**Key Clinical Message:**

Hepatic micro‐abscesses can be a rare initial presentation of systemic lupus erythematosus (SLE). This case highlights the importance of considering autoimmune etiologies when infectious causes are ruled out and emphasizes the need for early recognition and appropriate treatment of atypical hepatic manifestations in SLE to achieve favorable outcomes.

**Abstract:**

Systemic lupus erythematosus (SLE) is an autoimmune disease that affects multiple organs, including the liver. While hepatic involvement in SLE is typically subclinical or associated with mild liver enzyme elevations, rare manifestations such as hepatic micro‐abscesses and hepatic vasculitis have been reported. We report the case of a 27‐year‐old female who presented with persistent high‐grade fever, bilateral exudative lymphocytic pleural effusion, hepatic micro‐abscesses, anemia, and lymphopenia. Despite extensive investigations and antibiotic therapy, the patient's condition continued to worsen. The diagnosis of hepatic vasculitis, a rare manifestation of SLE, was ultimately made based on clinical suspicion, positive autoimmune markers, and negative septic workup. The patient responded well to high‐dose corticosteroid therapy and intravenous immunoglobulin, with resolution of liver lesions and clinical improvement. Hepatic involvement in SLE is diverse, and atypical presentations can pose diagnostic challenges. Hepatic vasculitis, although rare, should be considered in SLE patients presenting with liver lesions. The management involves immunosuppressive therapy, and prompt diagnosis is crucial to prevent further vascular damage. Hepatic micro‐abscesses, another rare manifestation of SLE, are thought to result from immune complex deposition. The exact pathogenesis remains unclear. Hepatic micro‐abscesses can have both infectious and non‐infectious causes, and it is very important to rule out common microbial pathogens. Treatment focuses on managing the underlying SLE activity with immunosuppressive agents. This case highlights the diagnostic challenges and management considerations in atypical hepatic manifestations of SLE. Awareness of rare presentations and collaboration among multiple specialties are essential for accurate diagnosis and appropriate treatment.

## 1 INTRODUCTION

SLE is an autoimmune illness that causes chronic inflammation and autoantibody production.^1^ SLE usually affects the musculoskeletal, renal, skin, cardiovascular, hematological, and central nervous systems, but it can also cause enteritis, protein‐losing enteropathy, or small bowel obstruction.^1^, ^2^ Hepatocellular or cholestatic patterns in liver function tests indicate subclinical hepatic involvement in SLE.^3^ SLE rarely presents with hepatic micro‐abscesses.^4^, ^5^ SLE liver damage is commonly diagnosed one or more years after the initial diagnosis, indicating delayed hepatic involvement.^3^ Up to 60% of SLE patients have liver function abnormalities, and 24% have hepatitis symptoms.^1^, ^2^, ^3^ Many patients with lupus flares have elevated liver enzymes and hepatitis symptoms.^6^ 21% of SLE patients with high liver enzymes and hepatitis symptoms experience lupus flares.^6^ A retrospective study indicated that 19 of 45 SLE patients with abnormal liver enzymes may have had lupus hepatitis.^7^ These findings demonstrate the intricacy of hepatic involvement in SLE and the necessity of understanding the processes. Several variables may cause liver problems in SLE, although the specific cause is uncertain. Drug‐induced liver dysfunction, alcohol intake, congestive heart failure, infections, and metabolic disorders are common secondary causes of SLE biochemical abnormalities.^1^, ^2^, ^3^, ^4^, ^5^, ^6^, ^7^ Fatty liver, portal inflammation, cirrhosis, cholestasis, chronic active, chronic persistent, non‐specific reactive, granulomatous, nodular regenerative hyperplasia, hepatic infarction, and arteritis have also been reported.^1^, ^2^, ^3^, ^4^, ^5^, ^6^, ^7^ Granulomatous hepatitis is rare in SLE, occurring in only a tiny percentage of histologically proven liver disorders.^8^ Hepatic vasculitis, another rare SLE symptom, is inconsistently reported.^9^ Liver vasculitis can cause hypodense lesions on imaging, hepatic necrosis, or spontaneous rupture.^9^, ^10^ In this case report, the difficulties in diagnosing hepatic vasculitis are highlighted, and the importance of a thorough evaluation in SLE patients who present with hepatic abnormalities is emphasized.

## 2 CASE HISTORY/EXAMINATION

A 27‐year‐old unmarried Filipino female, who had been working as an office secretary in Qatar for the past 5 years, presented with a 5‐day history of worsening cough, daily high‐grade fevers, shortness of breath, and pleuritic chest pain. She denied any recent travel outside of Doha and had no sick contacts. She was a non‐smoker, did not consume alcohol or use recreational drugs, had no animal exposure or pets at home, and had never used raw milk. She had no significant past medical or surgical history, and there was no history of recurrent urinary tract infections or pelvic inflammatory disease. On physical examination, the patient was febrile, and her other vital signs were stable. Decreased air entry was noted in the bilateral lower lung zones, but she was otherwise clinically stable.

## 3 METHODS (DIFFERENTIAL DIAGNOSIS, INVESTIGATIONS, AND TREATMENT)

**FIGURE 1:**
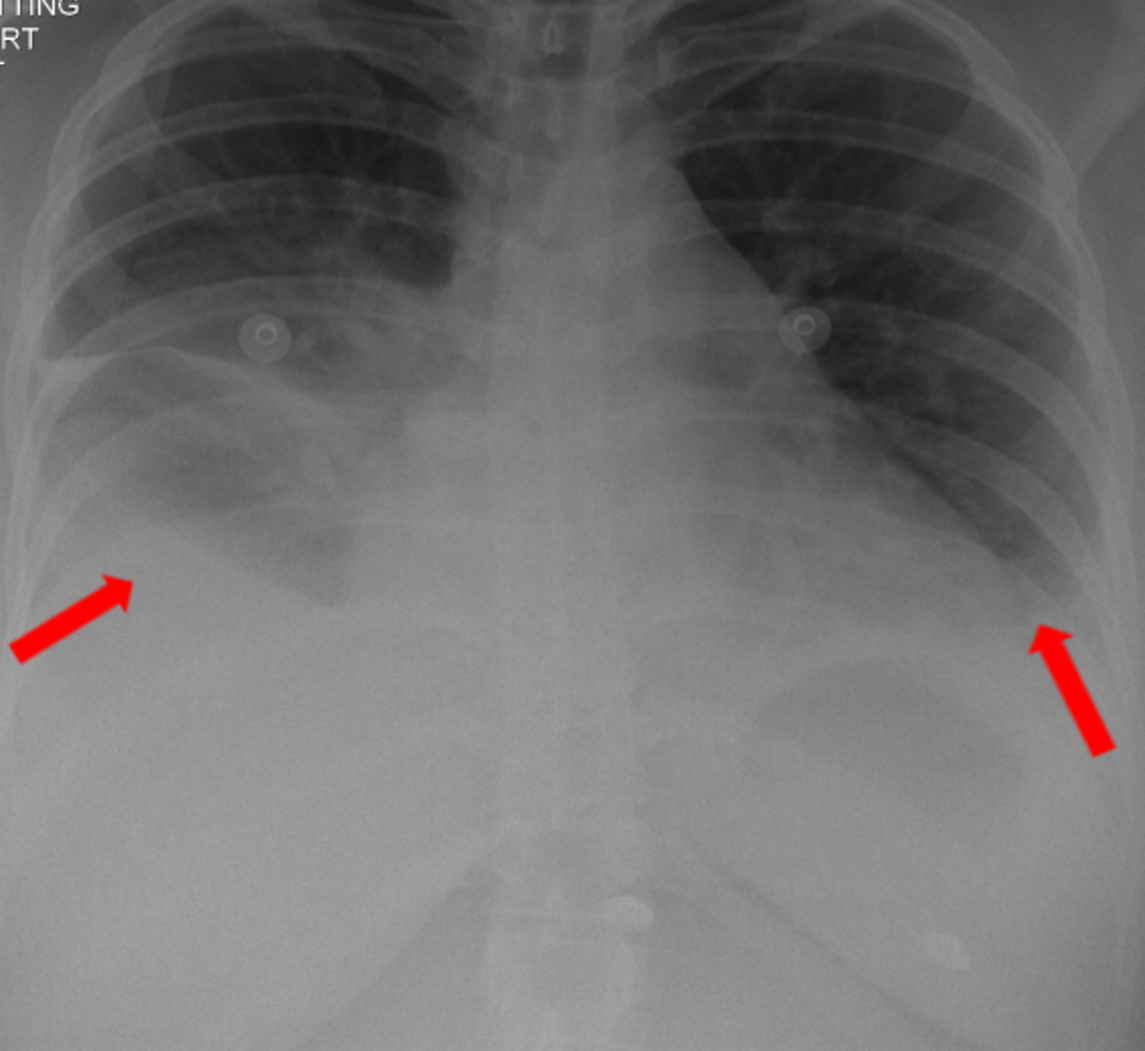
Chest radiograph AP view showing bilateral pleural effusion (Red arrows), with underlying collapse consolidation.

Initial laboratory investigations revealed normal renal function, liver function, electrolytes, and arterial blood gas levels. However, her C‐reactive protein (CRP) and procalcitonin were elevated (139 mg/dL, 2.5 ng/mL). Her complete blood count showed a low hemoglobin level of 9.7 g/dL, and the white blood cell count was within the normal range at 6.9 × 103 μL. The differential count showed an increased percentage of neutrophils (81.2%) and decreased lymphocytes (9.2%). A chest x‐ray revealed bilateral pleural effusion, more pronounced on the right side, with a possible underlying right‐sided collapse and consolidation ***(*** Figure 1 ***)*.** Based on the clinical presentation and investigations, the patient was admitted with a diagnosis of bronchopneumonia with parapneumonic effusion. She was started on intravenous piperacillin/tazobactam, which was later de‐escalated to ampicillin/sulbactam. Bedside pleural tapping was performed, and the pleural fluid analysis showed yellow, turbid fluid with an elevated protein level (56.0 g/L) and a predominant lymphocytic cell count. No organisms were seen in the sputum culture, pleural fluid culture or respiratory viral panel. After 5 days of hospitalization and intravenous antibiotics, she was discharged home with a prescription for oral augmentin to complete a total of 8 days of antibiotic therapy. She was also found to have anemia with low serum iron for which iron supplementation was initiated. However, 2 days after finishing the antibiotic course, the patient returned to the primary healthcare center with worsening cough, shortness of breath, persistent fever, and right pleuritic chest pain. A repeat chest x‐ray showed no definite pulmonary consolidation or masses but revealed blunting of both costophrenic angles, suggestive of bilateral pleural effusion. The patient was sent home with a five‐day course of azithromycin and reassurance.

Unfortunately, she returned to the emergency room a week later with further worsening symptoms. She required supplemental oxygen (oxygen saturation of 88% in room air, 94% on 3 L of oxygen via nasal canula), had tachypnea (a respiratory rate of 45/min), and tachycardia. Her blood pressure was normal, but breath sounds were decreased at the bilateral lung bases, and bilateral pleural effusion with underlying subsegmental collapse or consolidation was observed. Laboratory investigations during this admission showed worsening anemia (7 g/dL), lymphopenia (0.6 × 10^3^/μL), and thrombocytopenia (108 × 10^3^/μL). The CRP level was significantly elevated at 230, and procalcitonin dropped to 1.37. Renal and liver functions, as well as electrolytes, were within normal limits. Repeated pleural fluid analysis confirmed an exudative effusion with predominantly lymphocytic cells and no evidence of empyema or infectious organisms. A peripheral smear showed fewer red blood cells, mild lymphocytopenia, and a few neutrophils with toxic changes. There was worsening anemia with very low serum iron, low transferrin, low Total iron binding capacity (TIBC) and very high ferritin. All these features were suggestive of anemia of chronic diseases (ACD) and hence iron supplementation was stopped. Additional investigations, including a viral panel, tuberculosis, fungal workups, and malignancy workups, were all negative. Table 1 summarizes the important laboratory results during the patient's entire hospital course.

**TABLE 1:** A summary of important laboratory results during the entire hospital course.

Lab test (with units)	Value				Normal Range
	Initial Admission	Re‐admission (after 2 weeks)	Before starting immunosuppressive therapy	At 3‐month follow‐up	
WBC (×10^3^/μL)	6.9	5.6	6	9	4.0–10.0
Hgb (gm/dL)	9.7	7	6.8	10.3	12.0–15.0
Hct (%)	27.1	20.6	19.9	31	36.0–46.0
Platelet (×10^3^/μL)	269	108	79	306	150–410
Absolute neutrophil count (×10^3^/μL)	4.0	4.2	4.8	5.3	2.0–7.0
Lymphocyte (×10^3^/μL)	0.9	0.6	0.9	2.9	1.0–3.0
Urea (mmol/L)	3.2	6.4	7.3	2.2	2.5–7.8
Creatinine (μmol/L)	69	86	56	32	50–98
Sodium (mmol/L)	134	130	132	139	133–146
Potassium (mmol/L)	3.6	4	4.2	3.9	3.5–5.3
Chloride (mmol/L)	104.9	100	104	100	95.0–108.0
Bicarbonate (mmol/L)	23.4	20	25.2	27	22.0–29.0
Alanine amino transferase, ALT (U/L)	20	91	15	25	0–55
Aspartate amino transferase, AST (U/L)	22	129	35	24	5–34
C‐ Reactive protein (mg/L)	139	230	220	7.3	0–5
Procalcitonin (ng/mL)	2.5	1.39	1.5	0.21	<0.5
Serum iron (μmol/L)	<2	<2		19	9.00–30.40
Transferrin (gm/L)		1.56		2.6	2–3.6
Total iron binding capacity (TIBC) (μmol/L)		39		65	45–80
Ferritin (μg/L)		1373		89	12–114

**FIGURE 2:**
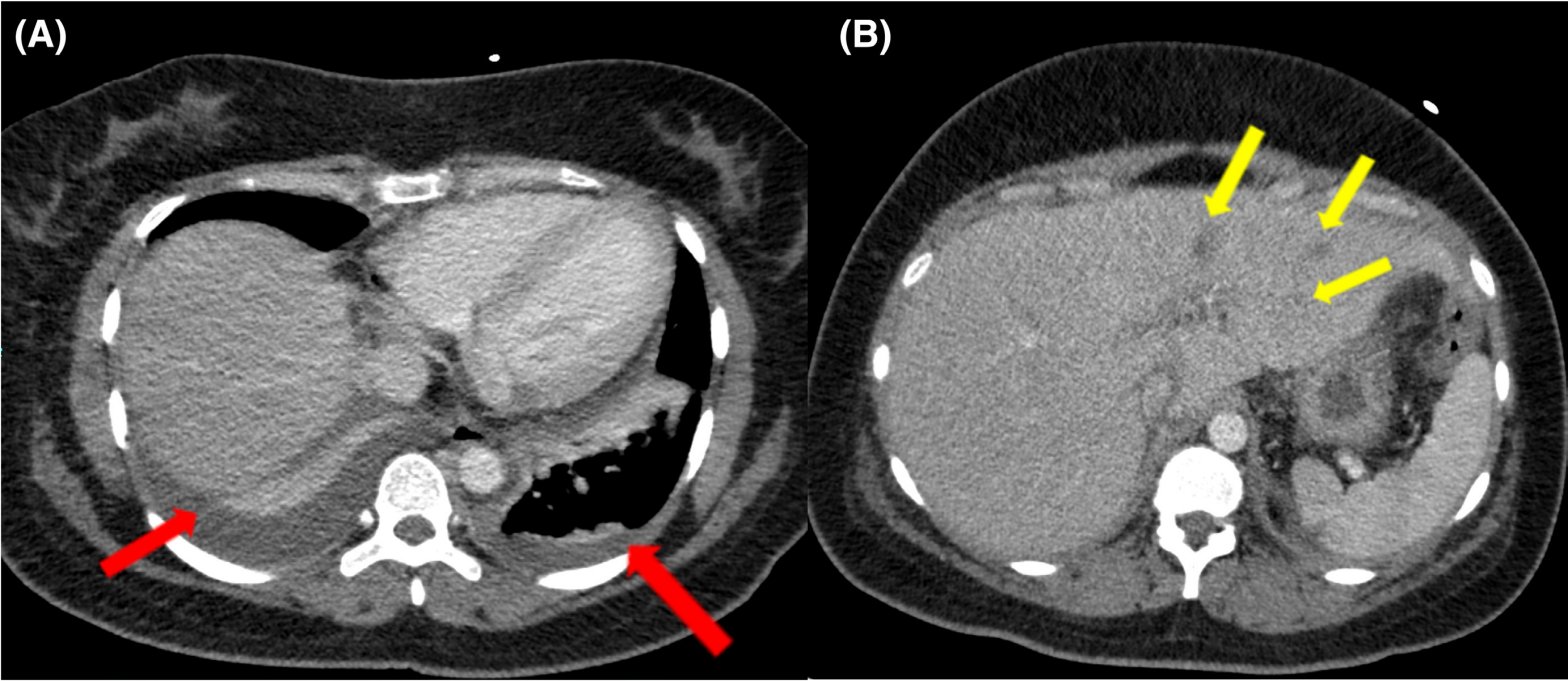
CT chest with intravenous contrast (A) Axial, mediastinal window cuts show bilateral pleural effusion (Red Arrows) with underlying collapse consolidation. (B) Axial, upper abdominal cut demonstrates multiple, small, ill‐defined hypodense foci in the left lobe of liver (Yellow arrows).

**FIGURE 3:**
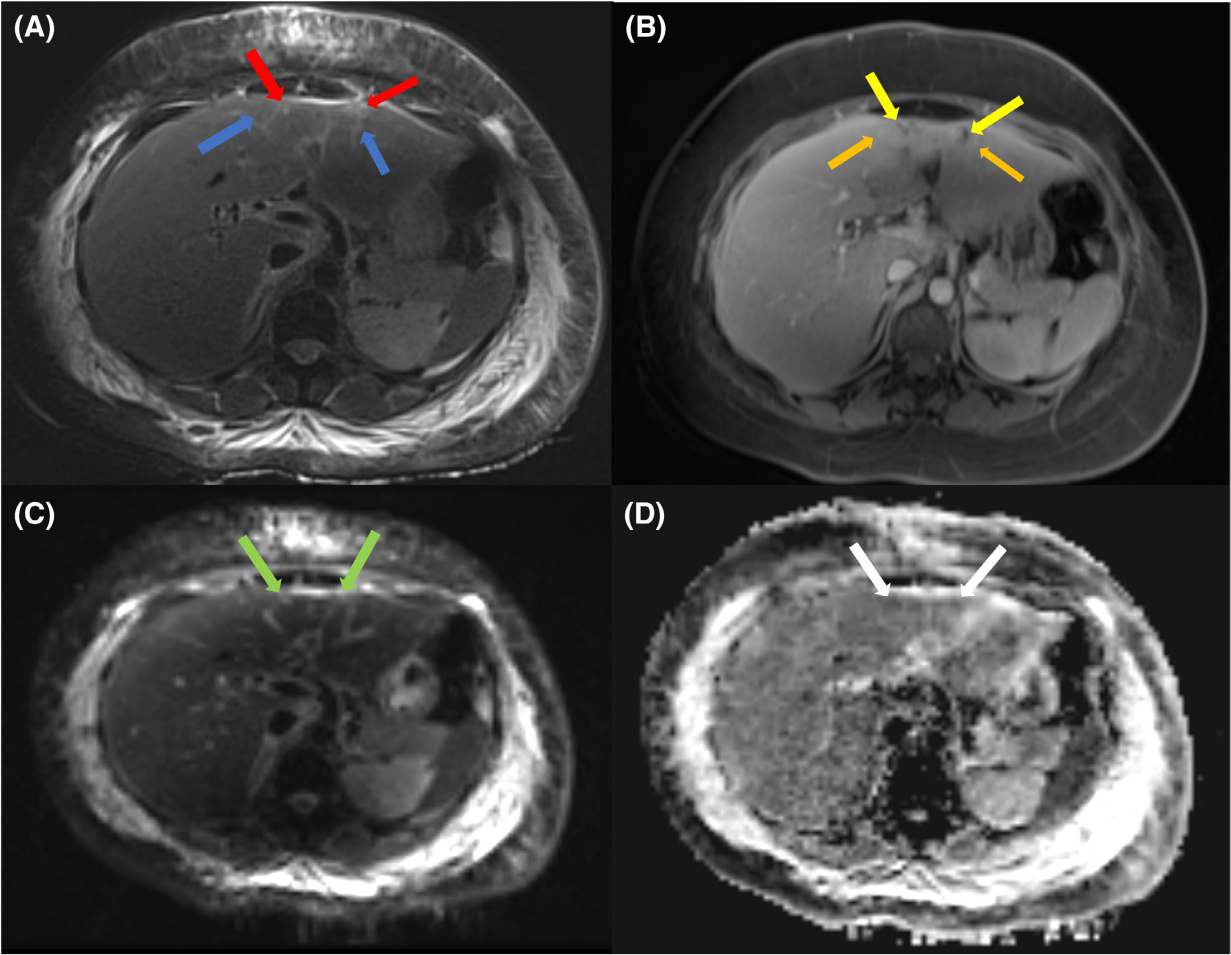
MRI Abdomen with intravenous contrast (A) T2 blade fat sat, (B) T1 post contrast venous phase, (C) DWI and (D) ADC, demonstrating T2 subcapsular linear high signal intensities in the left hepatic lobe (Blue arrows) which show heterogenous enhancement (Orange arrows). Multiple small T2 bright clusters (Red arrows) are seen, which are non‐enhancing on T1 post contrast (Yellow arrows). The small clusters of foci show high signal on DWI (Green arrows) and low signal on ADC (White arrows), suggestive of diffusion restriction.

Further imaging studies were conducted to investigate the cause of the persistent symptoms. An echocardiogram showed no evidence of pericardial effusion, intracardiac masses, or vegetation. CT scans of the thorax and abdomen revealed mild to moderate pleural effusion on the right side, mild pleural effusion on the left side with underlying collapse‐consolidation, and multifocal hypodense areas in the liver, suggesting evolving abscesses (Figure 2). MRI of the abdomen showed an enlarged liver with diffuse fatty infiltration. Abnormal T2 subcapsular linear high signal intensities in the left hepatic lobe with corresponding heterogenous enhancement and multiple non‐enhancing clusters of foci showing diffusion restrictions were noted. No corresponding intrahepatic biliary tree dilatation was seen (Figure 3). These findings were highly suggestive of hepatic micro abscesses. Multiple sets of blood cultures, pleural fluid cultures, and microbial investigations were repeated during fever spikes, but all remained negative. Abdominal ultrasound revealed tiny hypoechoic foci in the left lobe of the liver, possibly microabscesses. Considering the persistent fever, bilateral exudative lymphocytic pleural effusion, hepatic microabscesses, anemia, and lymphopenia, an autoimmune etiology was suspected. The patient's laboratory results showed positive anti‐nuclear antibodies (ANA) at a titer of 1:1280 with a speckled pattern, anti‐double‐stranded DNA (dsDNA) antibodies at a titer of 40, anti‐Ro antibodies greater than 240, and hypocomplementemia (Table 2). Additionally, lupus anticoagulant was positive, and the direct Coombs test was positive without evidence of frank hemolysis. The 24‐h urine protein level was 0.71 g (normal value is less than 100 mg per day). Based on these findings, the patient was diagnosed with systemic lupus erythematosus (SLE) with mainly serositis (bilateral pleural effusion, mild pericardial effusion) and possible lupus nephritis. Intravenous pulse steroid therapy with methylprednisolone (MP) at a dose of 500 mg daily was initiated for 3 days. Intravenous immunoglobulin (IVIG) was also administered at a dose of 2 g per kilogram over 4 days. The patient was then transitioned to maintenance steroids, starting with oral prednisolone at a dose of 40 mg daily. Hydroxychloroquine (HCQ) at a dose of 400 mg daily was also initiated. During the course of treatment, antibiotics were discontinued due to the lack of response and the suspected autoimmune etiology.

**TABLE 2:** Auto‐immune panel showing extra nuclear antigens (ENA) that favors a diagnosis of systemic lupus erythematosus (SLE).

Detail	Value w/Units	Normal range
Anti‐nuclear antibody	Positive, 1:1280, speckled	
Anti dsDNA antibody	Positive	
Antineutrophilic cytoplasmic antibody (ANCA)	Negative	
Anti‐RO antibody	Positive	
Anti‐LA antibody	Negative	
c3 (gm/L)	0.26	0.90–1.80
C4 (gm/L)	0.02	0.10–0.40
Anti cardiolipin antibody IgG	Negative	
Anti cardiolipin antibody IgM	Negative	
Anti B2 glycoprotein antibody IgG	Negative	
Anti B2 glycoprotein antibody IgM	Negative	
Rheumatoid factor (IU/mL)	Less than 10	0–14
Circulating anticentromere antibody (CENP)	Negative	
Anti‐Jo‐1 antibody	Negative	
Anti‐ribosomal P protein	Negative	
Anti ribo nucleo protein (RNP)	Negative	
Anti Scl‐70 antibody	Negative	
SmD antibody	Negative	

## 4 CONCLUSION AND RESULTS (OUTCOME AND FOLLOW‐UP)

**FIGURE 4:**
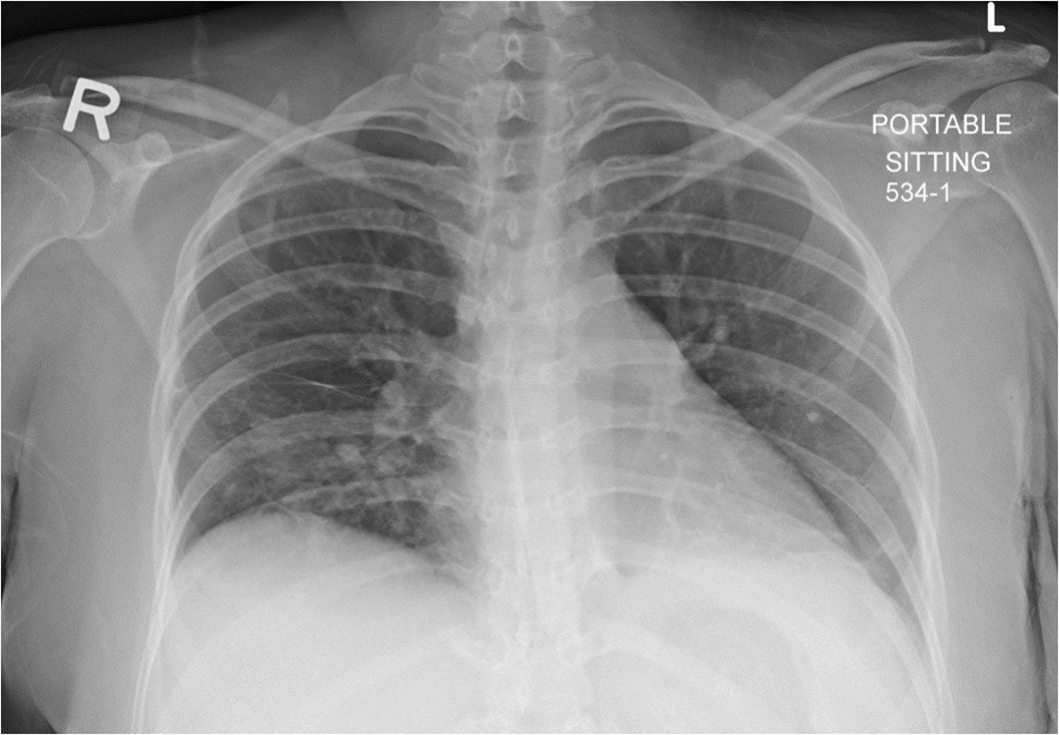
Chest radiograph showing complete resolution of the previously seen bilateral pleural effusion with some bibasilar atelectatic changes.

**FIGURE 5:**
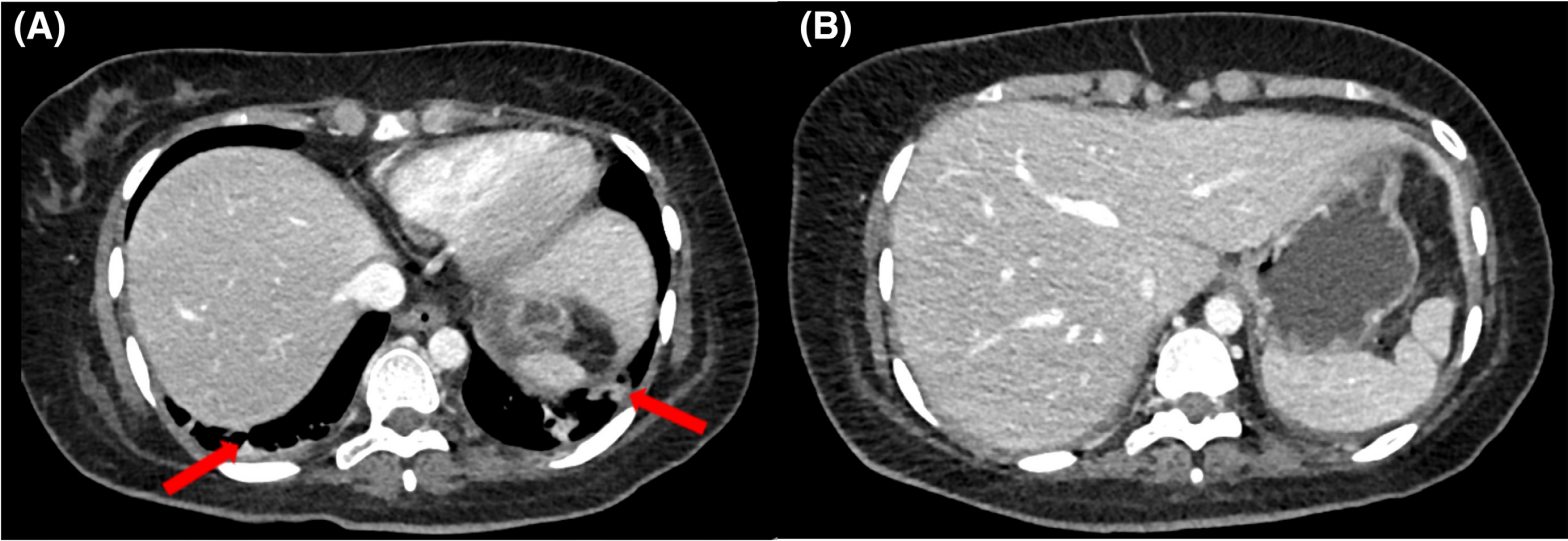
CT Abdomen with contrast, axial cuts (A) lower cut of thorax showing bilateral basal atelectasis with segmental collapse and complete resolution of the bilateral pleural effusion. (B) Total resolution of the previously seen micro abscesses in the left lobe of the liver was also noted.

A follow‐up chest x‐ray (Figure 4) and CT abdomen with intravenous contrast (Figure 5) after 6 weeks showed complete resolution of the bilateral pleural effusion and hepatic microabscesses. The patient was maintained on hydroxychloroquine 400 mg daily and prednisolone 5 mg daily, which were later tapered down and stopped. There was a significant improvement in laboratory values during follow‐up at 3 months (Table 1). A follow‐up after 6 months showed near complete resolution of clinical symptoms.

A conclusive diagnosis of hepatic involvement in this case is supported by the key features mentioned below:

- Imaging: CT and MRI scans revealed hypodense areas in the liver consistent with evolving micro‐abscesses
- Laboratory: Elevated inflammatory markers, anemia, lymphopenia, and positive autoimmune markers suggestive of SLE were observed. Negative results from infectious investigations supported a non‐infectious etiology.
- Clinical Presentation: Persistent fever, respiratory symptoms, and pleuritic chest pain persisted despite antibiotic therapy, hinting at an underlying systemic condition contributing to hepatic involvement.
- Exclusion of Other Causes: Absence of risk factors for infectious hepatic abscesses and negative infectious workups supported autoimmune etiology.
- Response to Treatment: Positive response to immunosuppressive therapy with resolution of hepatic micro abscesses on follow‐up imaging after intravenous pulse steroid therapy and immunoglobulin administration.

## 5 DISCUSSION

This case presents a diagnostic challenge due to the atypical presentation, poor response to initial therapy, and absence of a definitive diagnosis. The combination of bilateral exudative lymphocytic pleural effusion, hepatic microabscesses, anemia, lymphopenia, and persistently negative cultures raises concerns about an unusual infectious etiology, immune dysfunction, or an underlying systemic disease. Despite extensive investigations and antibiotic therapy, the patient's condition continued to deteriorate. This case underscores the importance of considering rare or atypical causes and emphasizes the need for comprehensive diagnostic evaluation to guide appropriate management in similar presentations. Further studies are warranted to determine the underlying etiology and optimize treatment strategies for such challenging cases. Systemic Lupus Erythematosus (SLE) is a complex autoimmune disease that can affect multiple organ systems, including the liver.^1^ Although overt liver disease is rare in SLE, subclinical manifestations or biochemical abnormalities in liver function tests are relatively common.^2^ Various hepatic manifestations have been described in SLE, including granulomatous hepatitis and hepatic vasculitis.^8^, ^9^, ^10^ This case report presents a patient with SLE who presented with multiple liver lesions that initially resembled abscesses but were ultimately attributed to hepatic vasculitis.

In retrospective studies, liver function abnormalities have been found in a significant proportion of SLE patients, and the diagnosis of liver disease often occurs after the diagnosis of SLE.^1^, ^2^ Hepatic vasculitis, although rare, has been reported in a small number of SLE cases.^4^, ^5^, ^9^ However, there is conflicting data on the incidence of hepatic vasculitis in SLE due to variations among different studies.^1^, ^2^, ^3^, ^4^, ^5^, ^6^, ^7^, ^8^, ^9^, ^10^ In this case, the patient presented with multiple liver lesions that resembled abscesses. The initial differential diagnosis included pyogenic liver abscesses and other infectious etiologies. However, due to clinical suspicion, positive autoimmune markers, and a negative septic workup, a diagnosis of hepatic vasculitis was considered. The patient was started on high‐dose corticosteroid therapy, which resulted in a dramatic clinical response and resolution of liver lesions on follow‐up imaging. This case highlights the diagnostic challenge of unusual hepatic manifestations in SLE patients. Hepatic vasculitis can present with various radiological findings,^11^ emphasizing the importance of maintaining a high index of suspicion for early diagnosis and appropriate management. Biopsy confirmation may be required to differentiate hepatic vasculitis from other causes and guide treatment decisions. In this case, the absence of infectious features, the presence of autoimmune thrombocytopenia, and the patient's response to corticosteroid therapy supported the diagnosis of hepatic vasculitis. The management of hepatic vasculitis in SLE involves immunosuppressive therapy to control disease activity and prevent further vascular damage.^4^, ^5^, ^9^ High‐dose corticosteroids are commonly used as the initial treatment, with the addition of other immunosuppressive agents if necessary.^4^, ^5^, ^9^ In this case, pulse steroid therapy, intravenous immunoglobulin, and hydroxychloroquine were administered, leading to clinical improvement and the resolution of liver lesions. Micro‐abscesses are tiny inflammatory collections that frequently result from pyogenic organisms within the liver parenchyma.^12^ In the context of SLE, these micro‐abscesses are thought to result from immune complex deposition and subsequent inflammation.^4^, ^5^ The exact pathogenesis of hepatic micro‐abscesses in SLE remains unclear, but it likely involves the interaction between immune complexes, complement activation, and impaired clearance mechanisms.^4^, ^5^ The differential diagnosis for hepatic micro‐abscesses includes infectious etiologies such as bacterial, fungal, or parasitic infections, as well as non‐infectious causes like SLE and other autoimmune diseases.^12^ In this case, an extensive infectious workup ruled out common microbial pathogens. Additionally, the absence of constitutional symptoms and negative blood cultures further supported the diagnosis of SLE‐associated hepatic micro‐abscesses. It is crucial to consider SLE as a differential diagnosis in patients presenting with unexplained hepatic lesions, particularly in the presence of suggestive clinical features and positive autoantibody markers. The treatment of hepatic micro‐abscesses in SLE involves managing the underlying SLE activity. Immunosuppressive therapy, including corticosteroids, hydroxychloroquine, and other immunosuppressive agents, forms the mainstay of treatment.^9^ Additionally, antimicrobial therapy targeting identified pathogens may be required in the presence of superimposed infections. Close monitoring of liver function tests, imaging studies, and clinical symptoms is crucial to assessing treatment response and detecting any potential complications.

Liver histopathological findings in SLE encompass a diverse range of lesions, often involving coexisting pathologies. It is essential to differentiate between liver involvement in SLE and other potential causes such as drug‐induced hepatitis, viral hepatitis, or autoimmune hepatitis (AIH). Common histopathological changes in the liver of SLE patients include steatosis, inflammation in portal tracts and lobules, granulomas, necrosis, microabscesses, hemosiderosis, cholestasis, and nonspecific reactive changes.^13^ Vascular changes, biliary changes, and advanced fibrosis or cirrhosis can also occur, albeit rarely.^13^ Differentiating between liver involvement in SLE and AIH can be challenging due to their similar clinical and serologic manifestations. Distinguishing features of AIH include portal and periportal inflammation, piecemeal necrosis, dominant portal tract plasma cell infiltration, and hepatocyte pseudorosette formation.^9^, ^13^ Lupus‐associated hepatitis predominantly exhibits lobular involvement with mild lobular inflammation and no piecemeal necrosis. The presence of antiribosomal P antibodies may help distinguish between the two conditions.^13^ Coexistent liver pathologies should be carefully considered and excluded in SLE patients. These can include drug‐induced hepatitis, viral hepatitis, primary biliary cirrhosis, granulomatous hepatitis, giant cell hepatitis, chronic hepatitis with immunoglobulin deficiencies, porphyria or idiopathic portal hypertension, and rarely lymphoma. The presence of these concurrent pathologies can alter the disease course and management.^9^, ^13^ The histopathological findings in the liver of SLE patients exhibit a wide morphological spectrum, often involving coexisting pathologies. It is crucial to exclude other potential causes, such as drug‐induced hepatitis or viral hepatitis, before attributing the changes solely to SLE. Common histopathological findings in SLE include a fatty liver, portal inflammation, and vascular changes. Having hepatic microabscesses as the first sign of systemic lupus erythematosus (SLE) is a rare occurrence, as this case report illustrates. When undiagnosed hepatic lesions are present, there should be a suspicion that SLE could be the underlying cause, especially if there are suggestive clinical features and positive autoantibody markers present. Achieving better results requires prompt identification and effective management of hepatic involvement in SLE. Through additional research, the pathogenesis of hepatic microabscesses in SLE must be clarified, and the best treatment approaches must be determined. Hepatic vasculitis is a significant SLE manifestation that can mimic other liver conditions, such as abscesses, despite being rare.

## AUTHOR CONTRIBUTIONS

**Sreethish Sasi:** Conceptualization; methodology; writing – original draft; writing – review and editing. **Mugahid Eltahir:** Writing – original draft; writing – review and editing. **Ezzeddin Ibrahim:** Writing – original draft; writing – review and editing. **Aishwariya Padmakumari:** Writing – original draft; writing – review and editing. **Jouhar Kolleri:** Writing – original draft; writing – review and editing. **Tasneem Abdallah:** Writing – original draft; writing – review and editing. **Ahmed Shehatta:** Writing – original draft; writing – review and editing. **Nawal Hadwan:** Writing – original draft; writing – review and editing. **Hani Jaouni:** Writing – original draft; writing – review and editing. **Muna Al‐Maslamani:** Writing – original draft; writing – review and editing.

## FUNDING INFORMATION

The publication of this article was funded by the Qatar National Library (QNL) through QNL open access program.

## CONFLICT OF INTEREST STATEMENT

The authors have no conflicts of interest to declare.

## ETHICS STATEMENT

The manuscript of this case report was approved by Institutional Review Board (IRB), Medical Research Centre (MRC) of Hamad Medical Corporation. Approval Number: MRC‐04‐23‐408.

## CONSENT

A written, and informed consent was obtained from the patient for publication of his case information and images.

## Data Availability

Data supporting the conclusions of the study is all available free of cost through open access journals and websites.
